# Vaccines for Leprosy and Tuberculosis: Opportunities for Shared Research, Development, and Application

**DOI:** 10.3389/fimmu.2018.00308

**Published:** 2018-02-26

**Authors:** Mariateresa Coppola, Susan J. F. van den Eeden, Naoko Robbins, Louis Wilson, Kees L. M. C. Franken, Linda B. Adams, Tom P. Gillis, Tom H. M. Ottenhoff, Annemieke Geluk

**Affiliations:** ^1^Department of Infectious Diseases, Leiden University Medical Center, Leiden, Netherlands; ^2^The National Hansen’s Disease Programs, Baton Rouge, LA, United States

**Keywords:** antigen 85B, early secretory antigenic target, *Mycobacterium leprae*, *Mycobacterium tuberculosis*, tuberculosis, leprosy, hybrid recombinant protein, vaccines

## Abstract

Tuberculosis (TB) and leprosy still represent significant public health challenges, especially in low- and lower middle-income countries. Both poverty-related mycobacterial diseases require better tools to improve disease control. For leprosy, there has been an increased emphasis on developing tools for improved detection of infection and early diagnosis of disease. For TB, there has been a similar emphasis on such diagnostic tests, while increased research efforts have also focused on the development of new vaccines. Bacille Calmette–Guérin (BCG), the only available TB vaccine, provides insufficient and inconsistent protection to pulmonary TB in adults. The impact of BCG on leprosy, however, is significant, and the introduction of new TB vaccines that might replace BCG could, therefore, have serious impact also on leprosy. Given the similarities in antigenic makeup between the pathogens *Mycobacterium tuberculosis* (*Mtb*) and *M. leprae*, it is well possible, however, that new TB vaccines could cross-protect against leprosy. New TB subunit vaccines currently evaluated in human phase I and II studies indeed often contain antigens with homologs in *M. leprae*. In this review, we discuss pre-clinical studies and clinical trials of subunit or whole mycobacterial vaccines for TB and leprosy and reflect on the development of vaccines that could provide protection against both diseases. Furthermore, we provide the first preclinical evidence of such cross-protection by *Mtb* antigen 85B (Ag85B)-early secretory antigenic target (ESAT6) fusion recombinant proteins in *in vivo* mouse models of *Mtb* and *M. leprae* infection. We propose that preclinical integration and harmonization of TB and leprosy research should be considered and included in global strategies with respect to cross-protective vaccine research and development.

## Introduction

Tuberculosis (TB) and leprosy are major infectious diseases that are caused by highly related mycobacterial pathogens, *Mycobacterium tuberculosis* (*Mtb*) and *M. leprae*. Although derived from the same mycobacterial ancestor (1), the target organs affected by these highly related mycobacteria (skin and nerves in leprosy; lungs and extrapulmonary lesions in TB) and the resulting clinical symptoms, are strikingly different. Notwithstanding these differences, the two poverty-associated diseases also share important characteristics (2–4), including the important role of host cellular immunity in protection. In addition, both diseases display a wide spectrum of (immuno)-pathological features with characteristic granulomatous lesions that often result in chronic disease and require prolonged treatment with multidrug antibiotic therapies (5).

Although rarely lethal, leprosy is enormously feared for causing lifelong handicaps and deformities resulting from irreversible nerve damage. Leprosy is notable for its continued transmission, which results in a stable annual number of approximately 200,000 new cases (6). Moreover, predictions from mathematical modeling indicate that millions linger undetected (7).

Tuberculosis is a major threat due to its high morbidity and mortality, causing an estimated 10.4 million new cases and 1.8 million deaths in 2015 alone (8). This scenario is worsened by HIV co-infection as well as by the emergence of multi-, extensive-, and total-drug resistance (8). Though not as threatening as for TB, anti-microbial resistance also poses a risk for leprosy (9–13), which needs to be considered in post-exposure prophylactic (PEP) treatment strategies in leprosy endemic areas that aim to reduce transmission by administering a single dose of antibiotics to those at high risk of developing leprosy (14).

In order to combat both diseases, global strategies have been endorsed, promoting the implementation of new drugs to shorten lengthy chemotherapeutic regimens, including strategies to avoid occurrence of *de novo* antibiotic resistance (15). In addition, research is focusing on development of improved diagnostics for detection of infection and early stages of disease allowing prophylactic and timely treatment, respectively. In contrast to chemoprophylaxis, vaccines would be expected to give rise to active as well as long-term protection. Therefore, development of novel vaccines is an additional top priority to control TB and leprosy by preventing disease and transmission (6, 16, 17). To explore this further, we here review the current vaccine development pipelines for TB and leprosy focusing on shared features and antigenic components, as well as highlight potential differences and incompatibilities.

## Bacille Calmette–Guérin (BCG), One Vaccine Fits All?

*Mycobacterium bovis*, BCG still is the only vaccine used against TB worldwide (18, 19). It is the first live-attenuated bacterial vaccine administered to newborns at or shortly after birth and has been applied in 172 countries (20, 21). In spite of its efficacy against severe TB in children, protection against TB in adolescents and adults is not sufficient to impact on disease and transmission. This urges for new, more efficient vaccines, and alternative strategies to replace or complement BCG (22–24).

Although being introduced and licensed for prevention of TB, BCG was soon recognized to protect partly also from leprosy (25–27). The efficacy of BCG vaccination against TB and leprosy has been evaluated in numerous clinical trials and observational studies. However, these studies also revealed inconsistent and sometimes even contradictory results. BCG’s protective effects varied from 2 to 83% and from 58 to 74% in preventing pulmonary and extrapulmonary TB, respectively (28), while its efficacy against leprosy ranged from 26 to 41% in experimental studies to 61% in observational studies, with mild differences between the paucibacillary (62%) and multibacillary (76%) forms (25, 29–31). BCG vaccination does not seem to protect against the third most common mycobacterial disease, Buruli ulcer’s disease, although a definite conclusion requires further well-designed prospective studies (32). Apart from its effect on mycobacterial diseases, BCG vaccination has been reported to have significant impact on unrelated diseases, probably through training of the innate immune system to respond more favorably to outer assaults (33, 34).

The remarkable differences in efficacy in various trials for TB and leprosy have been ascribed to several factors, including diversity in the genetic fingerprints of the mycobacterial pathogens in different geographic areas (35, 36), the various BCG strains used in the studies (37, 38), the immune, nutritional, and socioeconomic status of the vaccinees enrolled (39), the presence of helminths or viral coinfections (21, 40, 41), the background exposure to and induction of immunity by environmental mycobacteria, which might mask or block the effects of BCG (42), but the precise reasons for this remain largely unclear.

Our incomplete understanding of which components of the human immune system are responsible for either successful or inefficacious protection following BCG vaccination impedes the rational design of more effective vaccines (43). For instance, the limited efficacy of BCG in preventing local pulmonary TB disease compared to its effects on disseminated forms of TB is well documented, but remains unexplained (19). One hypothesis attributes this finding to its inability to induce durable and effective immune cells that home to the lung (19). Therefore, new routes of BCG administration, such as aerosol or intranasal immunization, are tested to initiate mucosal immunity and promote homing of immune cells to the lung mucosa (44, 45).

Another shortcoming of BCG is that its protective effects against TB as well as leprosy wanes over time, dropping to 14% efficacy after 10–20 years (46), indicating a suboptimal induction of long-term immune memory responses as discussed above (47, 48). Thus, BCG revaccination has been attempted in several countries. As a first attempt, a large trial in Malawi showed that BCG revaccination had limited impact on TB, while reducing the risk of leprosy with 50% (25, 49). Similarly, a large randomized controlled TB trial in Brazil showed that a second dose of BCG in adolescents did not confer better protection than a single dose given at birth (50). In contrast, for leprosy (30, 31), BCG revaccination is officially recommended in Brazil, since the 1970s for household contacts of leprosy patients as a boost to routine neonatal BCG vaccination. More recently, an extensive BCG revaccination trial of household contacts of leprosy patients in Brazil showed that the protection conferred by a booster BCG vaccination was 56% and was independent of previous BCG vaccination (29).

Notwithstanding, this lack of BCG boosting effects in TB and its beneficial effects on leprosy, BCG vaccination can also have less favorable effects, such as increasing the numbers of paucibacillary leprosy cases within the first months after BCG immunization (51). This is thought to be due to excessive boosting of pre-existing *M. leprae*-specific T cells in those already frequently exposed to the bacterium (51, 52), or to hyperinflammatory innate immunity (53, 54). Both mechanisms could lead to pathogenic immunity, such as increased numbers of paucibacillary leprosy and leprosy reactions (55).

Based on the premise that BCG might overcome the phenotypic cellular immunological tolerance against *M. leprae* in multibacillary leprosy, BCG immunotherapy has been trialed in leprosy patients in Venezuela in the 1980s (56). These studies met with limited success, since complications of this therapy were the occasional occurrence of disseminated cutaneous BCG lesions and the induction of leprosy reactional episodes (57). In contrast, a small-sized clinical trial in India studied a combination of MDT and immunotherapy with BCG in newly diagnosed leprosy patients and found a significant reduction in duration of reactions, incidence of type 2 reactions as well as in time to achieve bacterial clearance (58).

In summary, BCG has significant protective efficacy against severe TB in children and against leprosy in adults, while BCG revaccination has added value in leprosy, but not in TB. Future changes in TB vaccination policies might, therefore, also affect leprosy control. To further analyze this issue, we review current vaccine development pipelines and policies for TB and leprosy, focusing on shared target product profiles and antigenic composition.

## Vaccines in Clinical Trials: At the Crossroad Between Leprosy and TB

**Figure 1:**
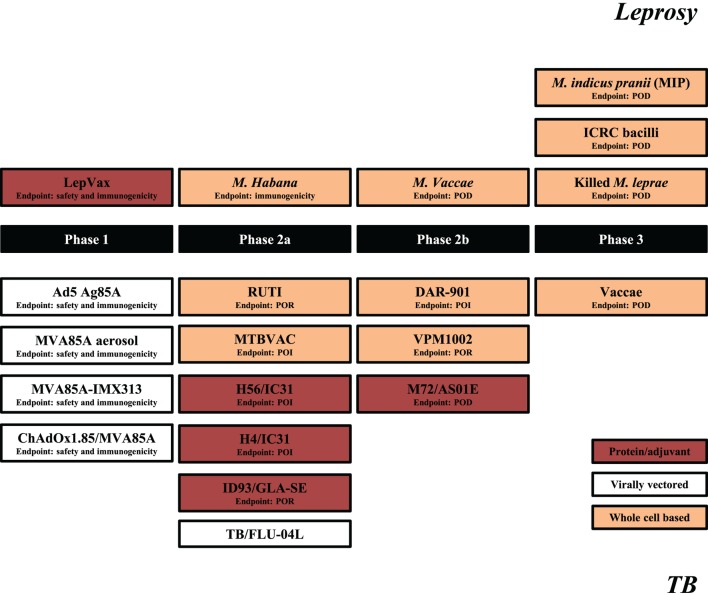
Leprosy and tuberculosis (TB) vaccine pipelines. Schematic representation of leprosy (upper segment) and TB (lower segment) candidate vaccines in clinical trials. Source: adapted from Ref. (59), TBVI/Aeras September 2017 and https://clinicaltrials.gov/. The primary endpoints are indicated for each trial with the exception of TB/FLU-04L for which primary outcome is yet not registered. POI, prevention of infection; POR, prevention of recurrence; POD, prevention of disease.

Although BCG vaccination trials in leprosy were executed decades ago, the current leprosy clinical vaccine pipeline is three times smaller than that of TB (Figure 1). This situation is relatively recent considering that in 2001 there were four candidate leprosy vaccines (being) tested in clinical trials vs. none against TB.

### Vaccine Candidates

The leprosy vaccine pipeline employs both live (26, 60) and killed (26, 56, 61–63) whole cell mycobacterial vaccines as well as adjuvanted recombinant protein vaccines, such as LepVax (64), which have the advantage over BCG and other replicating live vaccines that they can be used safely also in immunocompromised individuals (65). LepVax comprises a hybrid recombinant protein, linking four *M. leprae* antigens: ML2531, ML2380, ML2055, and ML2028 (LEP-F1) (Table 1), formulated in a stable emulsion with a synthetic, TLR4 agonist (GLA-SE) as adjuvant which has recently finished pre-clinical testing (66). In line with the extent of the epidemic, the TB vaccine pipeline is much larger. This includes candidates using various delivery platforms, such as virally vectored vaccines (67–70), adjuvanted subunits vaccines (71–74), recombinant BCGs (75), genetically attenuated *Mtbs*, as well as heat killed whole mycobacterial cell-based vaccines (76–79) (Figure 1). Evidently, the TB subunit vaccine pipeline has focused on a limited number of candidate *Mtb* antigens, in particular: Ag85A, Ag85B, early secretory antigenic target (ESAT6), TB10.4, Rv1813, Rv2608, Rv3619–3620, Rv1196, and Rv0125 (Table 1).

**Table 1:** Homology between tuberculosis (TB) vaccine components and *Mycobacterium leprae* proteins.

*Mycobacterium tuberculosis* proteins		Identity		Homology		*M. leprae* orthologs	Vaccine candidate	References
Rv number	Gene name	Amino acid (aa) overlaps	%	aa overlaps	%			
Rv3804c	Antigen 85A (Ag85A)	273/329	83%	296/329	90%	ML0097	Ad5 Ag85A; MVA85A aerosol; MVA85A-IMX313; ChAdOx1.85/MVA85A; TB/FLU-04L	(64–67)

Rv1886c	Ag85B	269/324	84%	288/324	89%	ML2028	H56/IC31; H4/IC31; TB/FLU-04L; LepVax	(61)

Rv3875	Early secretory antigenic target	35/91	39%	61/91	68%	ML0049	H56/IC31	(68)

Rv2660	Rv2660						H56/IC31	(68)
Rv0288	TB10.4	68/96	71%	82/96	86%	ML2531	H4/IC31; LepVax	(61, 69)
Rv1813c	Rv1813c	nssf	nssf	nssf	nssf		ID93/GLA-SE	(70)
Rv2608	PPE42	65/156	42%	88/156	56%	PPE family^a^	ID93/GLA-SE	(70)
Rv3619c	EsxV	59/92	64%	74/92	80%	ML1056	ID93/GLA-SE	(70)
Rv3620c	EsxW	55/95	58%	73/95	76%	ML1055	ID93/GLA-SE	(70)
Rv1196	PPE18	173/419	41%	228/419	54%	ML1054^b^	M72/AS01E	(71)
Rv0125	PepA	250/358	70%	292/358	82%	ML2659	M72/AS01E	(71)
Rv1860	Apa	197/298	67%	218/298	74%	ML2055	LepVax	(61)
Rv0455c	Rv0455c	101/152	67%	113/152	75%	ML2380	LepVax	(61)

*^a^Accession number not known; nnsf, no significant similarity found*.

*^b^Pseudogene*.

### Clinical Endpoints

Leprosy and TB vaccines have different target product profiles and clinical endpoints to be considered in efficacy trials, e.g., prevention of infection (POI), prevention of disease (POD), or prevention of recurrence (POR) (80). POD require extensive longitudinal studies due to the long incubation times (years) in TB and leprosy (years-decades), and the limited incidence rates in most populations studied. For these reasons, alternative clinical trial designs have been developed using alternative biologically relevant endpoints, such as prevention of recurrence (POR) in cured TB patients, which evaluate whether relapse rates can be reduced by post-therapy vaccination; or shortening of treatment trials, which evaluate whether treatment length can be reduced by complementary immunotherapy with TB vaccines during the last phase of TB treatment. For leprosy, vaccines could be positioned to help preventing nerve damage in patients, since this clinical endpoint has a much higher frequency in leprosy patients, requires a shorter follow-up period and is a highly relevant endpoint in leprosy. New clinical trial designs with alternative endpoints will be important to accelerate the clinical evaluation of new vaccines for TB and leprosy, and signals detected in such studies can be validated in larger studies against classical endpoints, such as POD and perhaps POI.

### Clinical Trials

In most vaccination trials for leprosy, the protective effects of the tested new vaccine candidates were equivalent to that of BCG (81). Only in one study, vaccination with Indian Cancer Research Centre bacilli (an *M. lep*rae-related cultivable mycobacterium) and BCG plus killed *M. leprae* showed a twofold increased protection against leprosy compared to BCG alone (26). However, *M. indicus pranii* (MIP) (also known as *Mycobacterium w*.) induced protective efficacy below that of BCG. Notwithstanding this result, MIP was evaluated also in a second, large-scale, double-blind trial with a 9-year follow-up (62). In this study, the protective efficacy of MIP in vaccinated household contacts after 3 years was the highest ever reported against leprosy (68%) for a vaccine other than BCG. However, its protective effect dropped considerably after 6 (60%) and 9 (28%) years of follow-up. Despite these conflicting results, MIP is currently being evaluated both as prophylactic and therapeutic vaccine against leprosy in two high endemic districts in India (82) in combination with a single dose of rifampicin (SDR). This design is reminiscent of a previous randomized vaccine field trial in which BCG as well as SDR was provided to leprosy contacts (83).

For TB, several vaccines and vaccine approaches are being pursued, with no new TB vaccine approved, yet for use, since the introduction of BCG in 1921. The results from the recent MVA85A vaccine phase 2b efficacy trial, the first new TB vaccine tested in an efficacy trial, since BCG, showed no improved protection in BCG-vaccinated South African infants (84), despite being highly immunogenic in adults (85). Several trials are ongoing (Figure 1), with the first outcomes to become available in 2018.

### Correlates of Protection

Vaccine immunogenicity studies for both leprosy and TB vaccine candidates have mostly focused on their ability to induce type-1 cell-mediated immunity, particularly CD4+ Th cells releasing type 1 helper (Th1) cytokines. Indeed, Th1 immunity is widely considered to be key in controlling mycobacterial infections (86). HIV-induced CD4+ T cell deficiency, and genetic or acquired impairments in type 1 cytokine signaling (IL12-IFN-γ axis), all increase susceptibility to mycobacterial infection and progressive disease in humans and animal models (87–90). In leprosy, the presence of Th1 cytokines in lesions or in lepromin skin reactions has been related to better clinical prognosis and to localized rather than disseminating disease (91, 92). Furthermore, individuals that showed large local reactogenicity after intradermal BCG administration or lepromin injection are reported to have less risk for leprosy onset (93). Observation from a small Dutch cohort of BCG-vaccinated individuals showed that high skin inflammation responders had a larger amount of C-reactive protein in their sera than the low skin inflammation responders. In the same study, at 4, 8, and 12 weeks post-BCG vaccination, PBMCs of individuals with stronger local reactogenicity induced higher IFN-γ production after *in vitro* PPD stimulation than the one from the group with less local reaction to BCG (94). This suggests that skin reactogenicity after BCG vaccination causing local inflammation and systemic Th1 responses probably indicate protective immunity to mycobacteria. The failure of MVA85A against TB despite its induction of CD4+ Th1 immunity, the observation that BCG-specific CD4+ and CD8+ T-cell responses did not correlate with protection against TB disease in one study (95) together with the limited results achieved by current leprosy vaccines, clearly underline the need for a better understanding of the host mechanisms that are responsible for protection against both TB and leprosy. Several recent reports in animal models and humans have reported the involvement of other cell subsets in leprosy and TB (96, 97). Discovering these mechanisms may well prove to be a critical step for designing more effective vaccines.

Besides BCG, only MIP and killed *M. vaccae* have been clinically evaluated for both leprosy and TB, although in different trial designs and target populations. MIP has been tested for its putative therapeutic efficacy in tuberculous pericarditis (98) and as mentioned above for its protective efficacy against leprosy (26, 62). Killed *M. vaccae* has been assessed for its ability to prevent TB and leprosy disease in patients or contacts. However, the administration routes (intramuscular vs. oral vs. intradermal injection of *M. vaccae*) and the eligibility criteria for the recruitment in the two trials (inclusion or not of individuals with BCG scar; HIV-positivity; anti-mycobacterial therapy) were quite diverse, impeding direct comparison of the impact of *M. vaccae* vaccination on both diseases (63, 78).

## One Subunit Vaccine for Both TB and Leprosy?

With the exception of *M. habana* (60, 99), the majority of vaccines evaluated for both leprosy and TB were initially designed as TB vaccines, and only evaluated at a later stage for their potential in leprosy. Since *M. leprae* has undergone massive gene reduction (100), not all *Mtb* antigens that are potential targets for TB vaccines have corresponding homologs in *M. leprae*. The first examples are ID83/GLA-SE and ID93/GLA-SE, two recombinant fusion proteins, formulated with the TLR4L-containing adjuvant GLA-SE, and consisting of three *Mtb* proteins: Rv1813, Rv2608, and Rv3620, with the further addition of Rv3619 in ID93. The amino acid (aa) sequences of Rv3619 and Rv3620 are 58 and 64% identical to the respective *M. leprae* proteins (ML1056 and ML1055, respectively) (Table 1). Likely due to these similarities, both *Mtb* hybrid recombinant proteins were also recognized by blood from paucibacillary leprosy patients, although latent *Mtb* infection could have explained these findings as well. Furthermore, when injected subcutaneously these vaccines reduced *M. leprae*-induced inflammation and bacterial growth in mouse models of leprosy (65), suggesting that TB subunit vaccines might have efficacy also against leprosy.

In a similar approach, we have investigated another TB subunit vaccine candidate, consisting of two major secreted *Mtb* proteins: *Mtb* ESAT6 and *Mtb* Ag85B, both present in short-term *Mtb* culture filtrates (101, 102). Ag85B is highly conserved among mycobacterial species, probably due to its critical role in cell wall synthesis as a mycolyltransferase (103). ESAT6 is a secreted virulence protein mainly restricted to the *Mtb* complex organisms (104). Both antigens have been extensively studied in the TB field over the past three decades and proved to be strongly recognized by CD4 Th1-cells of TB patients and latently TB infected (LTBI) individuals (105). Demonstrated to be immunodominant during *Mtb* infection, the two recombinant proteins were fused into a recombinant hybrid protein, and adjuvanted with the Th1 inducing synthetic adjuvant IC31^®^. In several animal models, including mice, guinea pigs, and non-human primates, Ag85B-ESAT6/IC31 showed promising protective efficacy against TB disease (106, 107). Based on these results, the vaccine was progressed to human phase 1/2a trials (105, 108, 109). This work demonstrated the vaccine’s safety and its remarkable ability to induce long-lasting Th1-type immune reactivity in healthy or HIV-negative, mycobacterially naive individuals, LTBI, and BCG-vaccinated volunteers (109–111) even 3 years after the second vaccination.

In view of several characteristics, Ag85B-ESAT6 is an interesting candidate for leprosy as well. *Mtb* ESAT6 and Ag85B share 68 and 89% aa overlaps (homology according to pre-computed Tuberculist Blastp) with *M. leprae* homologs, ML0049, and ML2028, respectively (Table 1). These proteins are widely recognized by antibodies of multibacillary leprosy patients (112, 113), as well as by IFN-γ secreting cells from paucibacillary leprosy patients (64). We previously demonstrated T-cell cross-reactivity between *Mtb* and *M. leprae* ESAT6 in leprosy and TB patients (114). Moreover, a previous study showed that Ag85B overexpression in BCG significantly increased BCG’s protective efficacy against *M. leprae* (115). To further explore and compare the efficacy of Ag85B-ESAT6-based vaccines against TB and leprosy, we generated both *Mtb* Ag85B-ESAT6 and *M. leprae* Ag85B-ESAT6 and studied their *in vivo* efficacy in mouse models of *Mtb* and *M. leprae* infection.

### *Mtb* and *M. leprae* Ag85B-ESAT6-Based Vaccines: A Comparative Evaluation

**Figure 2:**
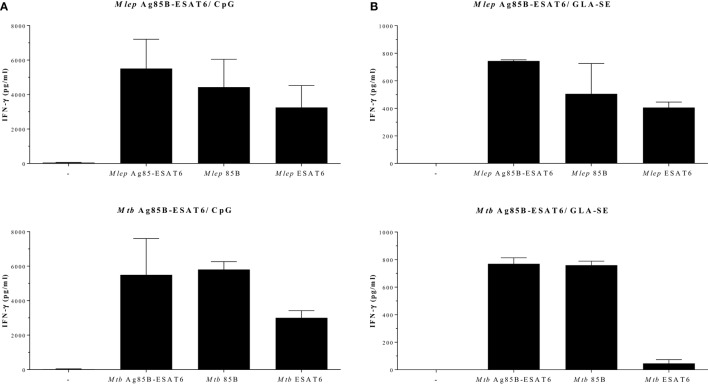
IFN-γ secretion after Ag85-ESAT immunization. C57BL/6j and HLA-A2tg mice B6.Cg-Tg (117) were purchased from The Jackson Laboratory (Bar Harbor, ME, USA) and housed under specific pathogen-free conditions. Recombinant proteins were overexpressed in *E. coli* BL21 (DE3) and purified to remove any traces of endotoxin as described in Ref. (116, 118). For the production of the antigen 85B (Ag85B)-early secretory antigenic target (ESAT6), hybrid recombinant hybrid protein, the Ag85B and ESAT6 genes were fused together by PCR with a linker coding for the amino acids NVA. C57BL/6j mice [**(A)**; 13–14 animals per group] and HLA-A2tg mice [**(B)**; 5 animals per group] were immunized three times subcutaneously with *Mycobacterium tuberculosis (Mtb)* Ag85-ESAT or *Mycobacterium leprae* Ag85-ESAT recombinant protein (25 µg) adjuvanted with GLA-SE [glucopyranosyl lipid adjuvant-stable emulsion (23) kindly provided by Infectious Disease Research Institute; Seattle, WA, USA; TLR4 agonist; 20 µg]; or CpG (ODN1826 5′-TCC ATG ACG TTC CTG ACG TT -3′; InvivoGen, San Diego, CA, USA; TLR9 agonist; 50 µg) (119). Splenocytes were harvested 4 weeks after final injections and restimulated *in vitro* with *Mtb* or *M. leprae* Ag85-ESAT hybrid recombinant proteins or the single Ag85B and ESAT6 recombinant proteins (all 10 µg/ml). IFN-γ secretion was analyzed by ELISA after 5 days. All mice were analyzed separately. Data shown indicate the mean and SE value of five mice per group.

**Figure 3:**
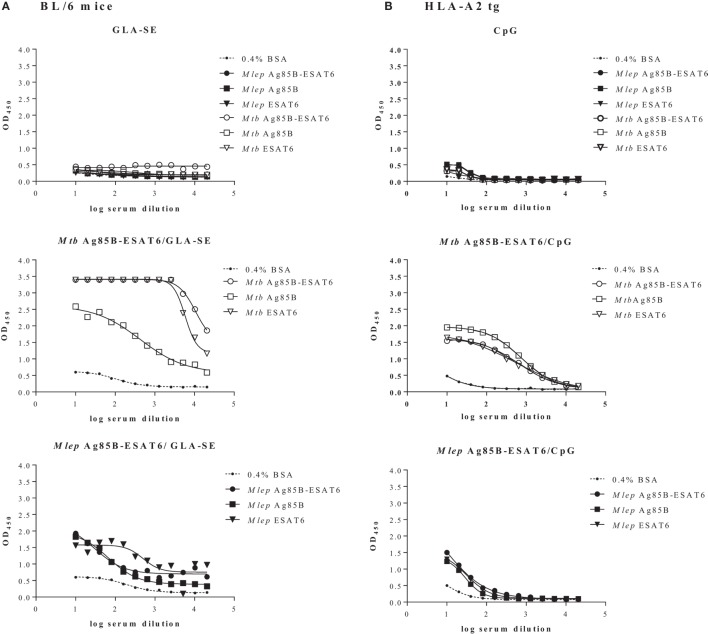
Quantification of serum antibodies. Following immunization of C57BL/6j **(A)** and HLA-A2 tg **(B)** mice with adjuvant alone, *Mycobacterium tuberculosis (Mtb)* Ag85-ESAT or *Mycobacterium leprae* Ag85-ESAT recombinant protein in GLA-SE **(A)** or CpG **(B)**, antibody titers (OD_450_) against *Mtb* Ag85-ESAT, *M. leprae* Ag85-ESAT, or *Mtb/M. leprae* Antigen 85B (Ag85B) and early secretory antigenic target (ESAT6) were determined by ELISA as described in Ref. (120). As a control coating with BSA (0.4% in PBS) was used. Sera from immunized mice were collected from cardiac blood 3 weeks after final immunization Serum dilutions are shown on the *x*-axis. Test groups included 3–5 mice. All mice were analyzed separately. Results are shown for one representative animal.

**Figure 4:**
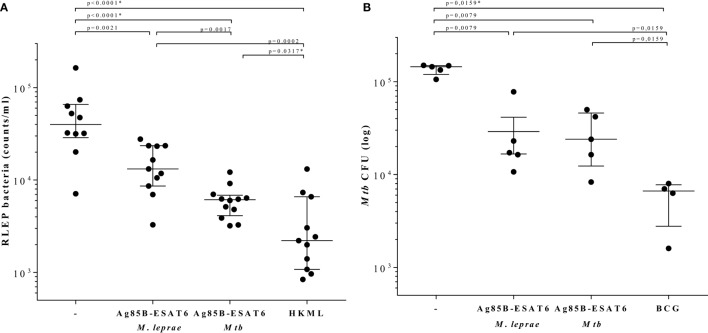
Determination of bacterial burden. C57BL/6j mice were injected with 10^4^ live *Mycobacterium leprae* (121) (viability: 11,000; in 40 µl PBS) in each hind foot pads 4 weeks after the final protein immunization. 7 months after *M. leprae* challenge, mouse footpads were harvested, and *M. leprae* were enumerated by RLEP PCR (122). HLA-A2tg mice were infected with live *Mycobacterium tuberculosis (Mtb)* strain H37Rv 6 weeks after the final protein immunization and 10 weeks after Bacille Calmette–Guérin (BCG) immunization (119). All animals included in the experiments were observed daily in order to ensure ethics requirements and to monitor any adverse effects possibly related to the vaccination or infection. **(A)** Bacteria were determined by the RLEP PCR from footpads from *M. leprae* infected C57BL/6j mice that had been immunized with GLA-SE adjuvant alone (−), *M. leprae* Ag85-ESAT/GLA-SE, *Mtb* Ag85-ESAT/GLA-SE, or heat killed *M. leprae* (HKML; 2 × 10^8^ in 40 µl; viability: 6,400) as indicated on the *x*-axis. Each symbol represents one mouse. Calculated bacterial loads are expressed as RLEP counts on the *y*-axis. Horizontal lines indicate median values with interquartile range. **(B)** CFUs were determined in lung homogenates from *Mtb*-infected unimmunized (−) or *Mtb*-infected HLA-A2 tg mice that were immunized with BCG1331 (10^6^ CFU), *M. leprae* Ag85-ESAT or *Mtb* Ag85-ESAT as indicated under the *x*-axis. Each symbol represents one mouse. Bacterial loads are expressed as log10 bacterial counts. Horizontal lines indicate median values with interquartile range. CFU of test and control groups were compared to the controls using the Mann–Whitney test and a *p* < 0.01 was considered significant. * marks differences that remained significant after multiple test correction using Kruskal–Wallis testing with Dunn’s post-test.

In order to evaluate the immunogenicity of *Mtb*-Ag85B-ESAT6 and *M. leprae*-Ag85B-ESAT6, both hybrid recombinant proteins were produced (116) and injected subcutaneously in wild-type C57BL/6j (BL/6) mice, as well as in C57BL/6j (BL/6) mice expressing an HLA-A*0201 transgene. The proteins were formulated with GLA-SE (TLR4 agonist) or CpG (TLR9 agonist), respectively, both of which have been reported to drive Th1-type responses. As expected, we detected high levels of IFN-γ released by splenocytes from immunized mice in response to *Mtb* Ag85B-ESAT6, *M. leprae* Ag85B-ESAT6, and their individual components (Figure 2). Total IgG, IgA, and IgM levels against *Mtb* Ag85B-ESAT6, *M. leprae* Ag85B-ESAT6, and the individual proteins were increased as well in both mouse strains following immunizations (Figure 3). Interestingly, the highest antibody titers were observed against *Mtb* Ag85B-ESAT6, regardless of whether *Mtb* Ag85B-ESAT6 or *M. leprae* Ag85B-ESAT6 had been used to immunize BL/6 mice (Figure 3A). Most importantly, both *Mtb* Ag85B-ESAT6/GLA-SE or *M. leprae* Ag85B-ESAT6/GLA-SE vaccines were capable of inducing host control of *Mtb* and *M. leprae* infection to a significant and comparable extent. Interestingly, *Mtb* Ag85B-ESAT6/GLA-SE controlled *M. leprae* infection significantly better than *M. leprae* Ag85B-ESAT6/GLA-SE (Figure 4). In summary, these results suggest that novel subunit vaccines designed for TB, such as *Mtb* Ag85B-ESAT6 could have efficacy against both TB and leprosy.

## Concluding Remarks

Leprosy and TB are still major poverty-related health concerns. Leprosy is primarily endemic in geographic areas, where TB is also highly prevalent (115). To date, BCG has been used predominantly as a vaccine against TB, but it also contributes to the control of leprosy. However, due to its limited efficacy especially against pulmonary TB in adults, the main and contagious form of TB, novel vaccines are being developed to replace or boost BCG (Figure 1). Although these vaccines will likely also impact leprosy incidence, this issue is rarely considered, let alone studied in extensive trials.

There are two leprosy vaccine candidates, MIP in India (82) and LepVax (66), and the TB vaccine pipeline is much more advanced and diverse than the one for leprosy. Even though it is likely that a TB vaccine candidate will emerge, for none of the current TB candidate vaccines, the impact on leprosy is currently being taken into account.

Only two highly similar recombinant subunit TB vaccines, based on the same backbone design, have been tested for their potential use against leprosy (65). Here, we describe original data showing a second TB subunit candidate vaccine platform, based on Ag85B/ESAT6. Collectively, our data suggest that novel TB vaccine candidates can cross-protect against leprosy, providing support for integrating leprosy vaccine research with TB vaccine research (65, 81, 115). At the moment, the most advanced new TB vaccine candidates have been tested in India, Tanzania, China, South Africa, the first two of which have elevated incidences of leprosy. Thus far, none of these recent trials have included evaluation of impact on leprosy, unlike what was done decades ago for BCG (61). We contend that preclinical integration and harmonization of TB/leprosy discovery and development research would well be feasible with respect to the design of subunit vaccines, as we have in fact applied in our recent approach for vaccine antigen discovery (123). With respect to antigen selection algorithms, it is of interest to consider the extensive genomic reduction that *M. leprae* has undergone during evolution (100, 124), causing this *Mycobacterium* to become a highly specialized and obligate intracellular pathogen (125). Studying *M. leprae*’s successful minimalistic approach will reveal genetic and metabolic pathways that pathogenic mycobacteria need to survive in the host, and inspire drug and vaccine efforts to combat both diseases which have put such a heavy toll on humans for millennia.

## Ethics Statement

The handling of mice was conducted in accordance with the regulations set forward by the animal care committee of the LUMC and in compliance with European Community Directive 86/609 for the care and use of laboratory animals. The experiments at NHDP were performed under a scientific protocol reviewed and approved by the NHDP Institutional Animal Care and Use Committee (Assurance #A3032-01) and were conducted in accordance with all state and federal laws in adherence with PHS policy and as outlined in The Guide for the Care and Use of Laboratory Animals, Eighth Edition.

## Author Contributions

Concept of the review: AG and TO. Designed and wrote the review: MC, TO, and AG. Figures and legends: SE, KF, NR, LW, and LA. Final approval of the version to be published: all authors.

## Conflict of Interest Statement

The authors declare that the research was conducted in the absence of any commercial or financial relationships that could be construed as a potential conflict of interest.
